# Supplementary material for: Sequential addition of cations increases photoluminescence quantum yield of metal nanoclusters near unity
Source: Nat Commun. 2025 Jan 11;16:587. doi: 10.1038/s41467-025-55975-y (PMC11724975; doi:10.1038/s41467-025-55975-y)
Supplement: Supplementary file 1 — Supplementary Information [file 41467_2025_55975_MOESM1_ESM.pdf]

Supporting Information

Sequential addition of cations increases photoluminescence quantum yield of metal nanoclusters near unity

Xue Wang,[1,§] Yuan Zhong,[1,§] Tingting Li,[2] Kunyu Wang,[1] Weinan Dong,[1] Min Lu,[1] Yu Zhang,[1] Zhennan Wu,[1,*] Aiwei Tang[3,*] and Xue Bai[1,*]

[1]State Key Laboratory of Integrated Optoelectronics, College of Electronic Science and Engineering, Jilin University, Changchun 130012, P. R. China

[2]College of Materials Science and Engineering, Jilin Jianzhu University, Changchun 130012, P. R. China

[3]Key Laboratory of Luminescence and Optical Information, Ministry of Education, School of Physical Science and Engineering, Beijing Jiaotong University, Beijing 100044, P. R. China

*Corresponding author: wuzn@jlu.edu.cn; awtang@bjtu.edu.cn; baix@jlu.edu.cn

[§]Xue Wang and Yuan Zhong contributed equally to this work.

Supplementary figures

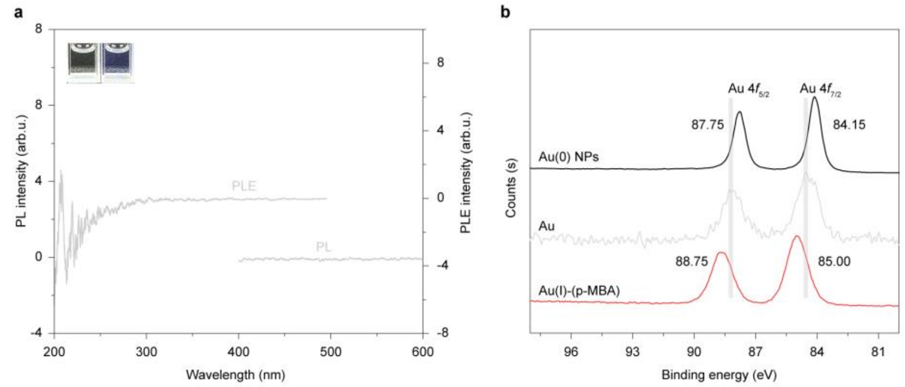

Supplementary Fig. 1 a PLE, and PL spectra of **Au** NCs (λ_{ex} = 365 nm). Insets are the photographs of the aqueous solution of **Au** NCs under visible light (left) and 365 nm-UV light (right). **b** High-resolution Au 4*f* XPS spectra of **Au** NCs. The gray lines represent the peak center of Au 4*f* XPS of **Au** NCs in **b**.

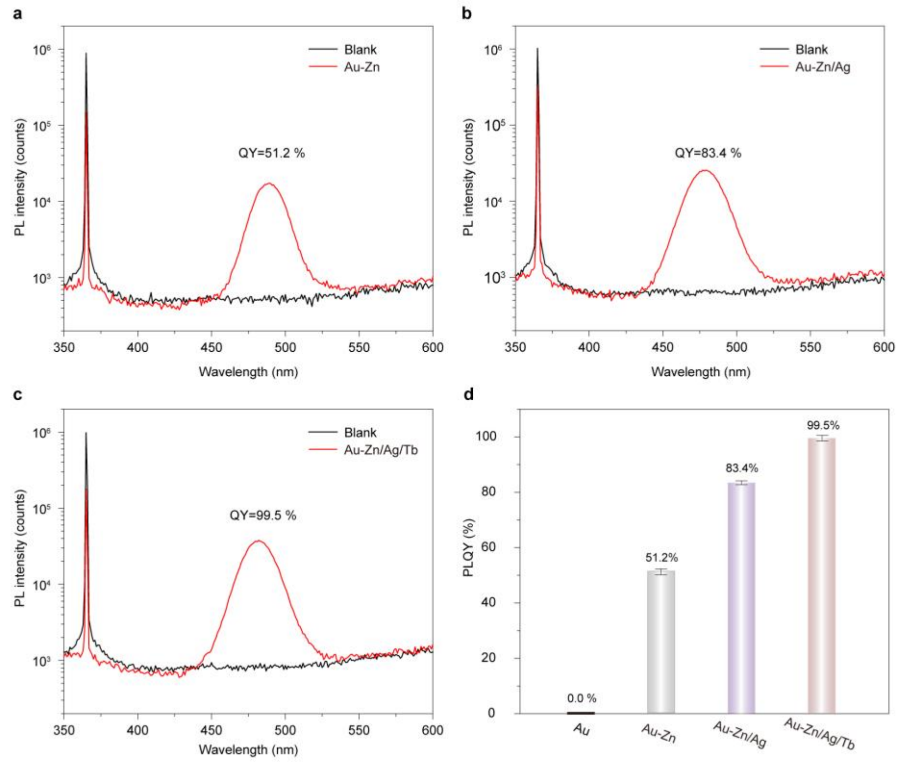

Supplementary Fig. 2 a-c Calculation of PLQY of the aqueous solution of **Au-Zn**, **Au-Zn/Ag**, and **Au-Zn/Ag/Tb** NCs, respectively. **d** The statistics of recorded PLQY values of **Au**, **Au-Zn**, **Au-Zn/Ag**, and **Au-Zn/Ag/Tb** NCs, respectively. The error bar is calculated as follows: error bar = mean value ± standard deviation, the number of samples is six.

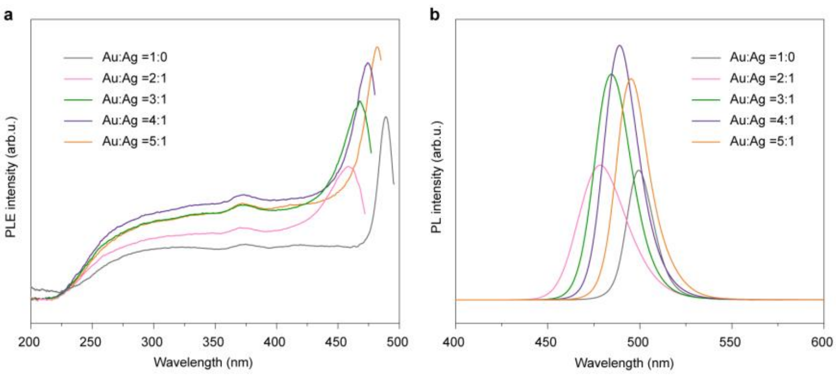

Supplementary Fig. 3 a PLE and **b** PL spectra of **Au-Zn/Ag** NCs added with different Au and Ag molar ratios.

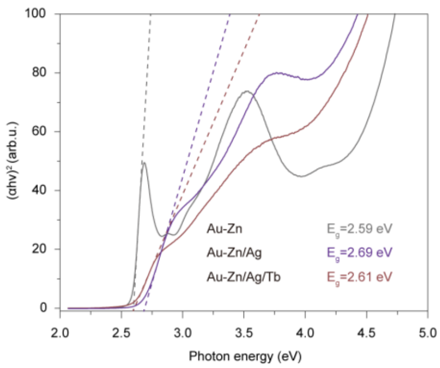

Supplementary Fig. 4 Calculation of optical bandgap energies of **Au-Zn**, **Au-Zn/Ag**, and **Au-Zn/Ag/Tb** NCs.

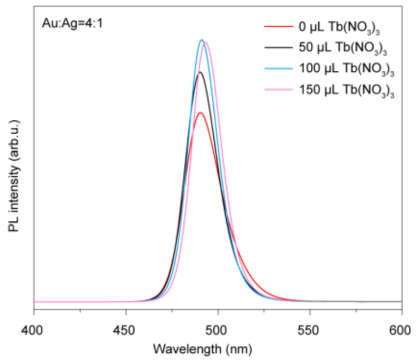

Supplementary Fig. 5 PL spectra of **Au-Zn/Ag/Tb** NCs added with different amounts of $Tb(NO_3)_3$.

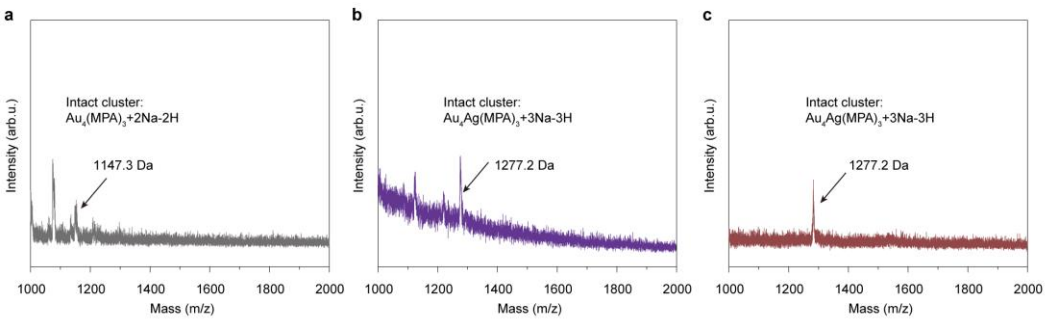

Supplementary Fig. 6 a-c Positive MALDI-TOF mass spectra of **Au-Zn**, **Au-Zn/Ag**, and **Au-Zn/Ag/Tb** NCs, respectively. DCTB was used as the matrix for the test of all samples. The maximally prominent peak was detected at 1147.3, 1277.2, and 1277.2 Da, corresponding to the mass signals of $Au_4(MPA)_3$ NCs, $Au_4Ag(MPA)_3$ NCs and $Au_4Ag(MPA)_3$ NCs. Other peaks in the mass spectrum with smaller mass values were assigned to the laser-induced fragmentations. In Supplementary Fig. 6a, a strong peak at 1083 Da is attributed to the signal of [$Au_4(MPA)_2$+Na+Zn-2H] fragment. A weak peak (1211.7 Da) on the right side of the 1147.3 peak is due to the adsorption of Zn^{2+} on the [$Au_4(MPA)_3$+2Na-2H] NCs and can be attributed to the [$Au_4(MPA)_3$+2Na+Zn-2H].

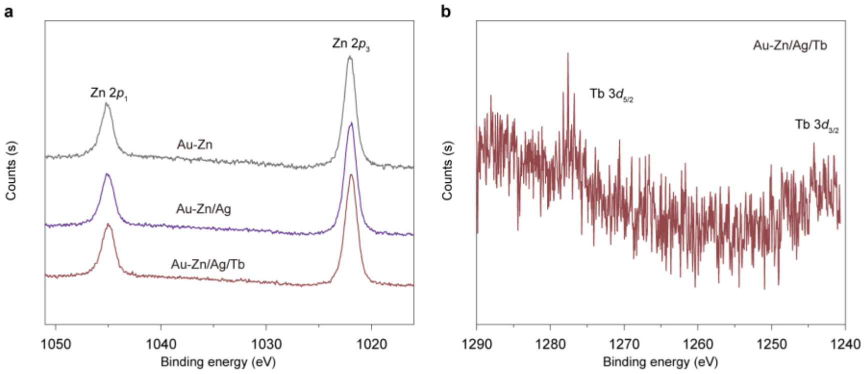

Supplementary Fig. 7 a High-resolution Zn 2*p* XPS spectra of **Au-Zn**, **Au-Zn/Ag**, and **Au-Zn/Ag/Tb** NCs, respectively. **b** Tb 3*d* XPS spectra of **Au-Zn/Ag/Tb** NCs.

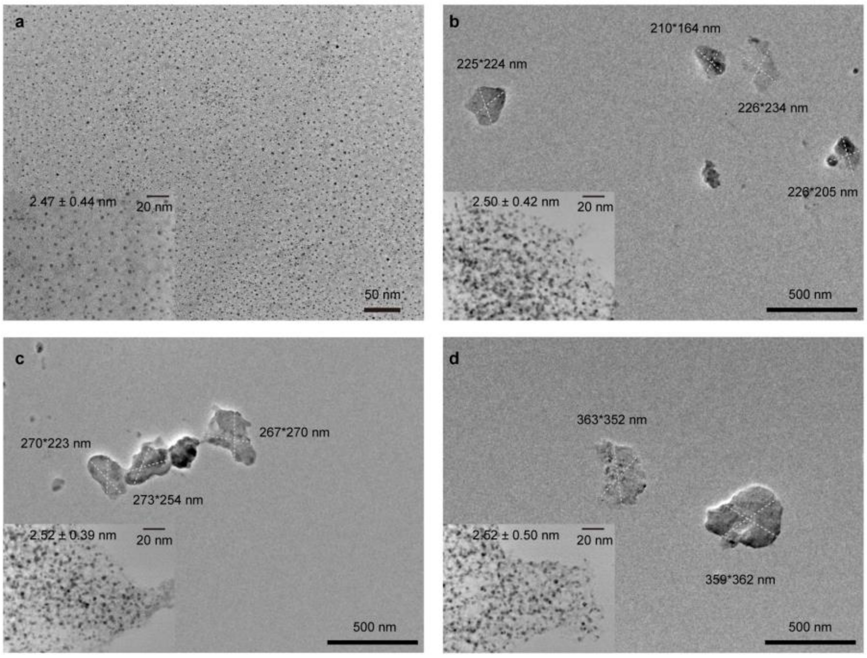

Supplementary Fig. 8 a-d TEM images of **Au, Au-Zn, Au-Zn/Ag, and Au-Zn/Ag/Tb** NCs, respectively. The insets are amplified images of the corresponding samples.

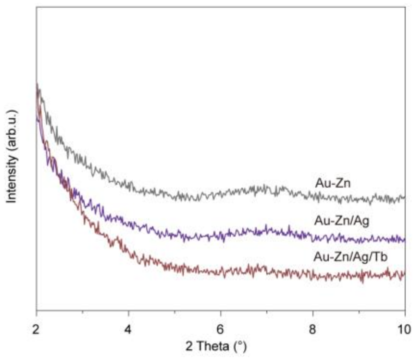

Supplementary Fig. 9 SAXRD spectra of **Au-Zn**, **Au-Zn/Ag**, and **Au-Zn/Ag/Tb** NCs.

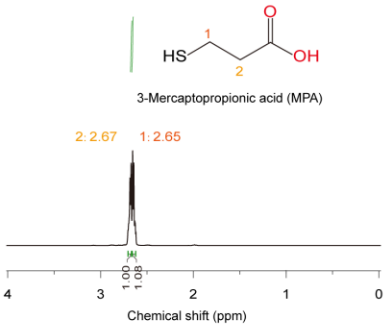

Supplementary Fig. 10 ^{1}H NMR spectra of MPA ligand in D_2O.

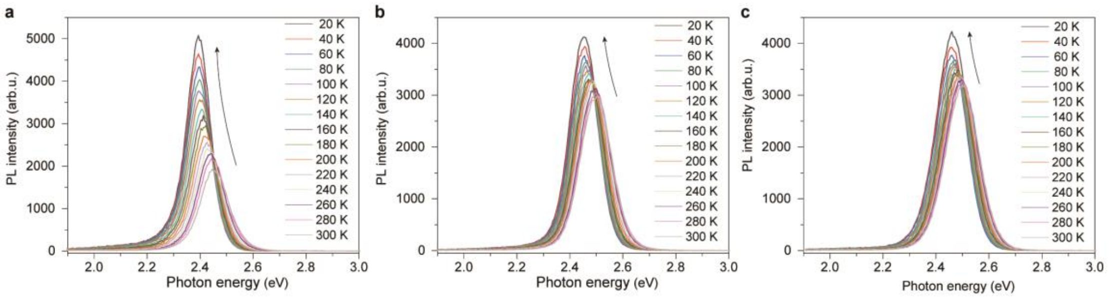

Supplementary Fig. 11 a-c Temperature-dependent PL spectra of **Au-Zn**, **Au-Zn/Ag**, and **Au-Zn/Ag/Tb** NCs, respectively. The excitation source is 365 nm light and the temperature range is 20-300 K with a temperature interval of 20 K.

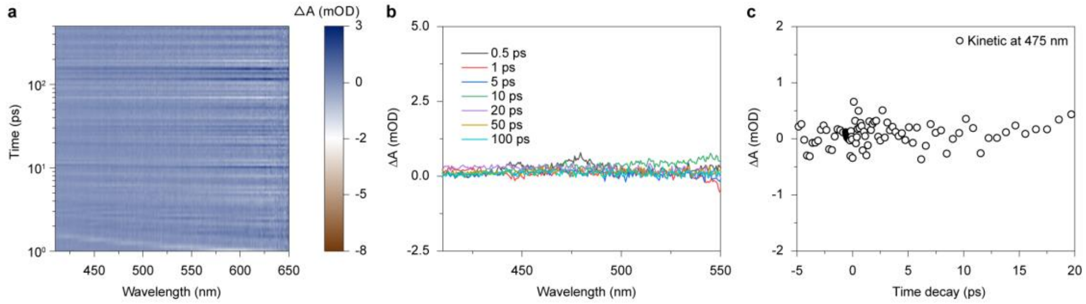

Supplementary Fig. 12 The fs-TA spectra and electron dynamics of isolated **Au** NCs. **a** The fs-TA map pumped at 365 nm. The scaling of the color scales is the absorption intensity, and the units are milli-optical density. **b** The fs-TA profile at different time delays. **c** The kinetic decay curve.

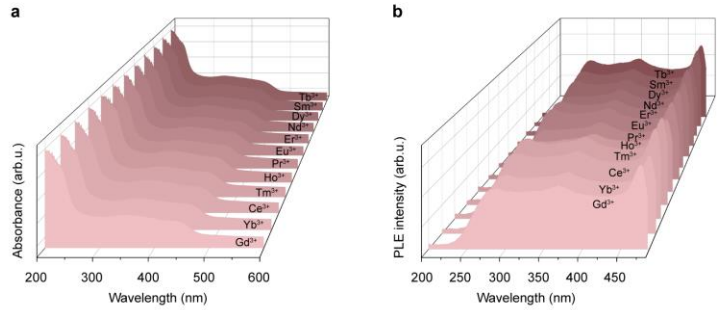

Supplementary Fig. 13 a UV-vis absorption spectra and **b** excitation spectra of **Au-Zn/Ag/R** NCs (λ_{ex} = 365 nm). "R" is for Ce^{3+}, Pr^{3+}, Nd^{3+}, Sm^{3+}, Eu^{3+}, Dy^{3+}, Ho^{3+}, Er^{3+}, Tm^{3+} and Yb^{3+}, respectively.

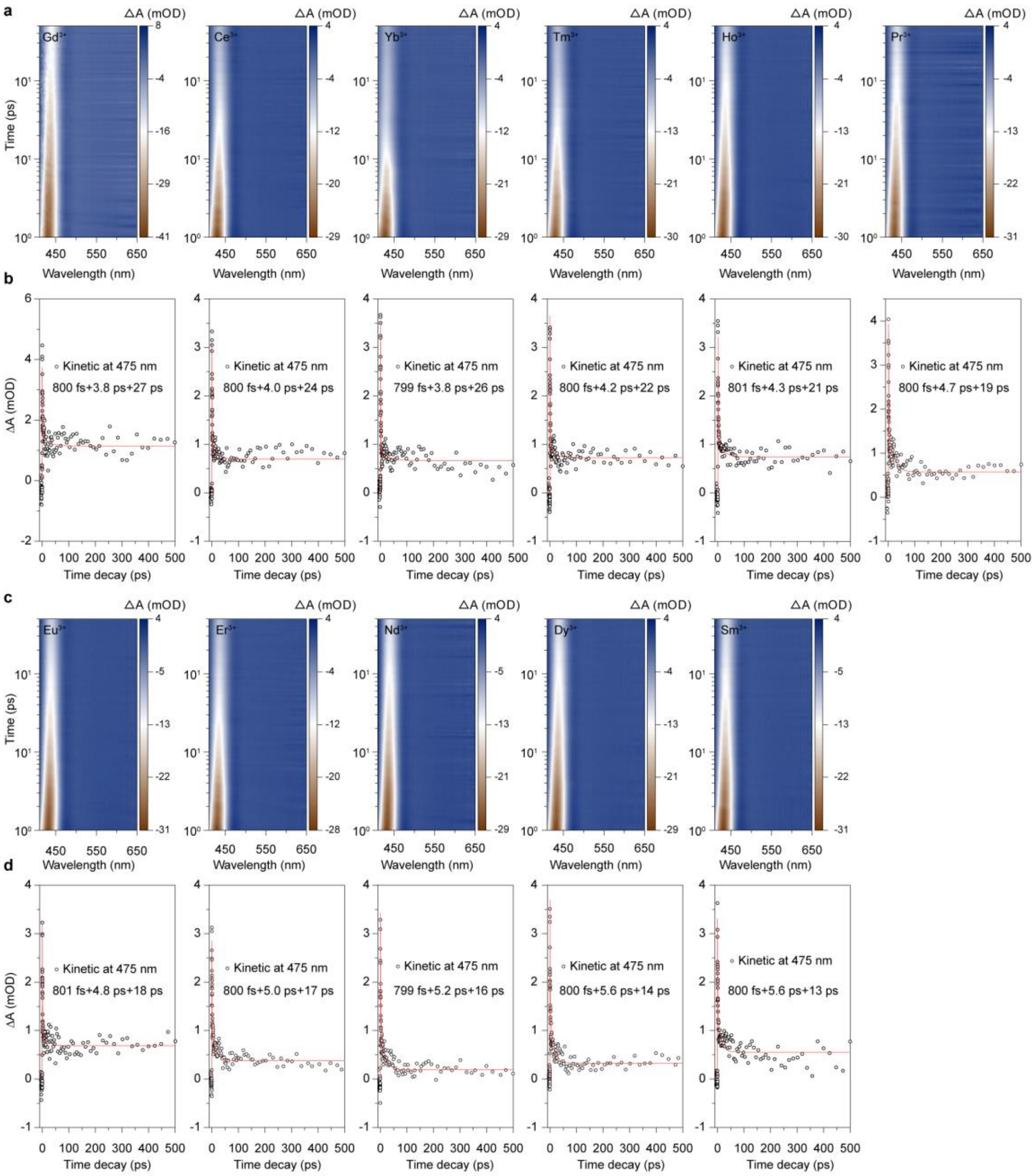

Supplementary Fig. 14 a,c The femtosecond-TA maps of **Au-Zn/Ag/R** NCs upon 365 nm laser excitation. The scaling of the color scales is the absorption intensity, and the units are milli-optical density. **b,d** The kinetic decay at 475 nm of **Au-Zn/Ag/R** NCs within 500 ps. "R" is for Ce^{3+}, Pr^{3+}, Nd^{3+}, Sm^{3+}, Eu^{3+}, Dy^{3+}, Ho^{3+}, Er^{3+}, Tm^{3+}, and Yb^{3+}, respectively.

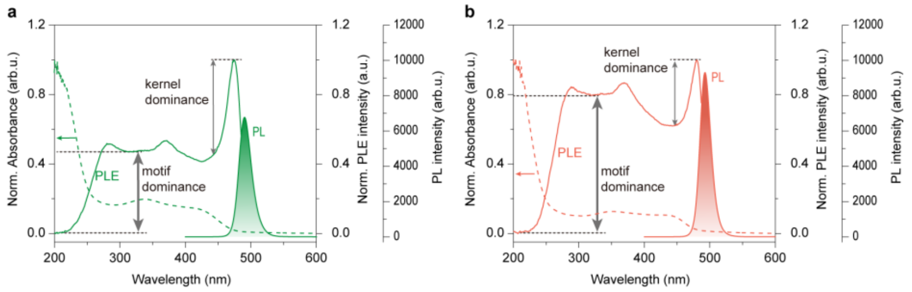

Supplementary Fig. 15 a,**b** UV-vis absorption, PLE, and PL spectra of **Au-Zn/Cu**, and **Au-Zn/Cu/Tb** NCs, respectively (λ_{ex} = 365 nm).

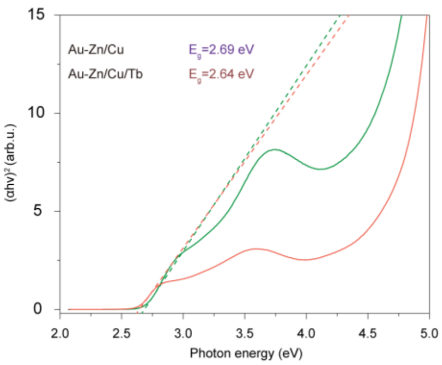

Supplementary Fig. 16 Calculation of optical bandgap energies of **Au-Zn/Cu**, and **Au-Zn/Cu/Tb** NCs.

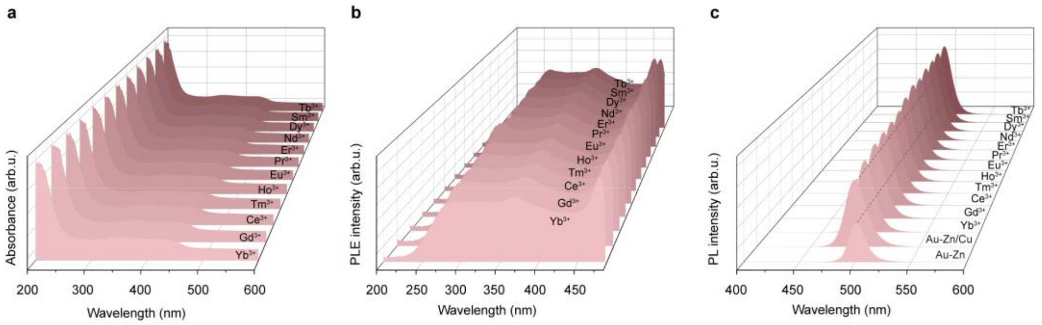

Supplementary Fig. 17 a UV-vis absorption, **b** PLE, and **c** PL spectra of A**u-Zn/Cu/R** NCs. (λ_{ex} = 365 nm). "R" is for Ce^{3+}, Pr^{3+}, Nd^{3+}, Sm^{3+}, Eu^{3+}, Dy^{3+}, Ho^{3+}, Er^{3+}, Tm^{3+} and Yb^{3+}, respectively.

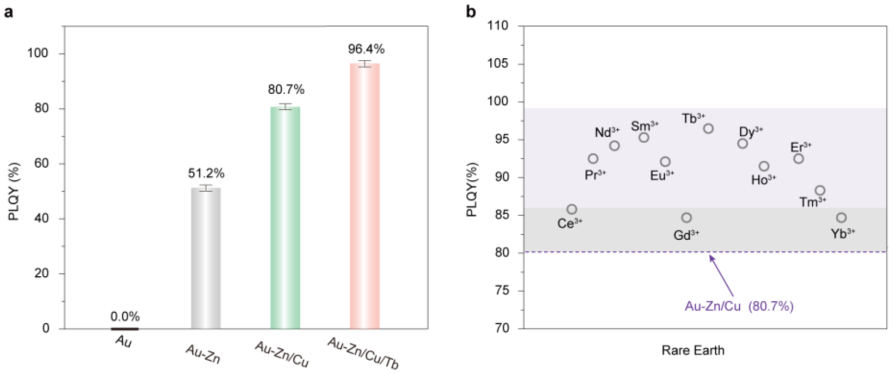

Supplementary Fig. 18 a The statistics of recorded PLQY values of **Au-Zn**, **Au-Zn/Cu**, and **Au-Zn/Cu/Tb** NCs, respectively. The error bar is calculated as follows: error bar = mean value ± standard deviation, the number of samples is six. **b** The comparison in PLQY between **Au-Zn/Cu** and **Au-Zn/Cu/R**. “R” denotes arbitrary rare earth ions, for instance, Ce^{3+}, Pr^{3+}, Nd^{3+}, Sm^{3+}, Eu^{3+}, Gd^{3+}, Dy^{3+}, Ho^{3+}, Er^{3+}, Tm^{3+}, and Yb^{3+}. The gray shaded area represents a similar PLQY for **Au-Zn/Cu/R** and **Au-Zn/Cu**. The reddish-brown shaded area represents different degrees of increase of the **Au-Zn/Cu/R** PLQY.

Supplementary tables

Supplementary Table 1 Optical parameters of **Au-Zn**, **Au-Zn/Ag**, and **Au-Zn/Ag/Tb** NCs.

Gold NCs	Absolute PLQY (%)	FWHM (meV)	Stokes shift (meV)
Au-Zn	51.2	86	61
Au-Zn/Ag	83.4	108	85
Au-Zn/Ag/Tb	99.5	99	75

Supplementary Table 2 Fluorescent lifetime components of **Au-Zn**, **Au-Zn/Ag**, and **Au-Zn/Ag/Tb** NCs obtained from TCSPC measurements.

Gold NCs	τ_1 (ns)	A	τ_2 (ns)	A	$\tau_{ave.}$ (ns)	χ^2
Au-Zn	18.4 （42%）	2834.0	38.9 （58%）	1828.8	30.2	1.1346
Au-Zn/Ag	22.3 （11%）	1038.7	43.5 （89%）	4108.1	41.1	1.1293
Au-Zn/Ag/Tb	43.6 （100%）	4953.7	-	-	43.6	1.0417

Supplementary Table 3 Fitting parameters of normalized TA in Fig. 4. Each decay possesses a different time constant. In this paper, the τ_1 component is ascribed to the internal conversion (IC) of hot electrons from S_n to the S_1 state ($S_n \rightarrow S_1$). τ_2 component comes from core-directed structural vibration in the series of NCs superstructures. τ_3 component can be assigned to the relevant electron transfer process from the excited state of staple motifs to the metal core.

Au-based NCs	A_1 ($\times10^{-3}$)	τ_1 (fs)	A_2 ($\times10^{-3}$)	τ_2 (ps)	A_3 ($\times10^{-3}$)	τ_3 (ps)	Reduced χ^2 ($\times10^{-3}$)
Au-Zn	7.358	800±1.8	12.57	3.1±0.4	10.08	40±0.5	0.19
Au-Zn/Ag	6.759	800±1.5	1.424	3.8±0.3	1.787	27±0.3	1.43
Au-Zn/Ag/Tb	2.441	800±1.6	0.141	5.6±0.4	0.454	12±0.4	1.61
